# Risk-Benefit Assessment of Feed Additives in the One Health Perspective

**DOI:** 10.3389/fnut.2022.843124

**Published:** 2022-02-10

**Authors:** Alberto Mantovani, Gabriele Aquilina, Francesco Cubadda, Francesca Marcon

**Affiliations:** Istituto Superiore di Sanità - National Institute of Health, Rome, Italy

**Keywords:** risk-benefit assessment (RBA), feed additives, one health (OH), iodine, cobalt, aflatoxin M1, mycotoxin binders, formaldehyde

## Abstract

Safety and sustainability of animal feeds is a pillar of the safety of the entire food chain. Feed additive assessment incorporates consumer safety as well as animal health and welfare, which, in turn, can affect productivity and hence food security. The safety of feed users and the environment are other important components of the assessment process which, therefore, builds on a One Health perspective. In several instances the assessment entails a balanced assessment of benefits and risks for humans, animals and/or the environment. Three case studies are selected to discuss issues for a consistent framework on Risk-Benefit Assessment (RBA) of feed additives, based on EFSA opinions and literature: (a) Supplementation of feeds with trace elements with recognized human toxicity (cobalt, iodine) - RBA question: can use levels, hence human exposure, be reduced without increasing the risk of deficiency in animals?; (b) Aflatoxin binders in dairy animals - RBA question: can the use reduce the risk for human health due to aflatoxin M1, without unexpected adverse effects for animals or humans?; (c) Use of formaldehyde as preservative in feedstuffs to prevent microbial contamination - RBA question: is the reduction of microbiological risks outweighed by risks for the consumers, farmed animals or the workers? The case studies indicate that the safety of use of feed additives can involve RBA considerations which fit into a One Health perspective. As in other RBA circumstances, the main issues are defining the question and finding “metrics” that allow a R/B comparison; in the case of feed additives, R and B may concern different species (farm animals and humans). A robust assessment of animal requirements, together with sustainability considerations, might be a significant driving force for a RBA leading to a safe and effective use.

## Introduction

Safety and sustainability of animal feeds is a pillar of the safety of the entire food chain (1–4). A wide number of substances may be present in farm animal feeds depending on the feed composition for the different animal species and categories as well as the origin and quality of feed ingredients (1). Besides feed components, a wealth of different substances are added to feeds in order to protect and promote the health, welfare and productivity of farm animals. However, environmental xenobiotics and natural undesirable substances may also be present and their impact has to be concurrently assessed.

**Figure 1:**
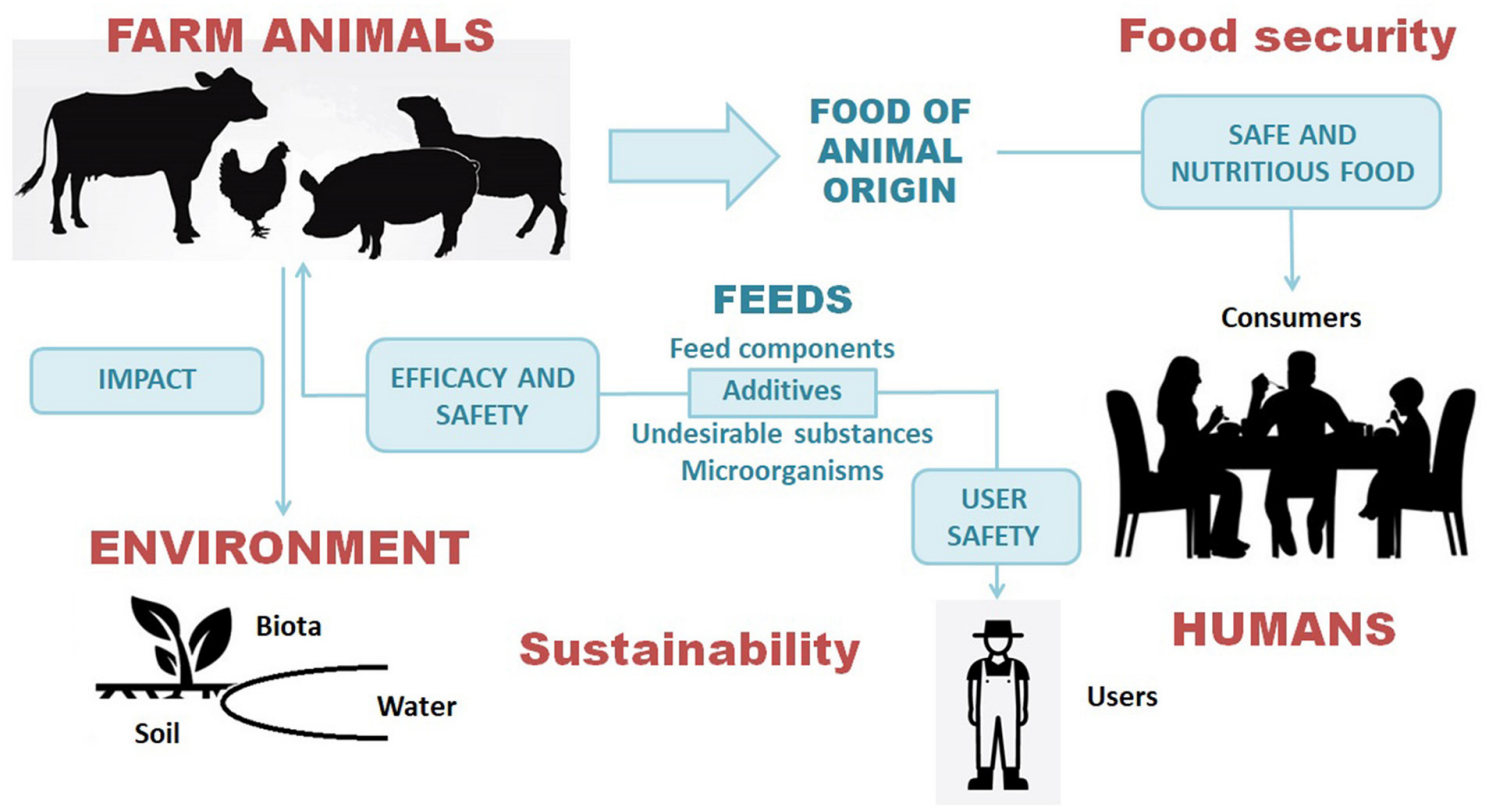
Safety and sustainability of animal feeds as a pillar of the safety of the entire food chain in a One Health perspective: relationships between animal health and welfare, consumer safety, safety of feed users and the environment.

Along with the obvious economic advantages for animal farming, a correct use of feed additives may increase the availability of safe, nutritious and sustainable foods of animal origin, hence supporting human food security (Figure 1): meanwhile, scientific evidence should support the proposed conditions of use of each active substance (5). The European Union food safety framework is based on the “farm to fork” concept. Thus, the risk assessment of feed additives is pivotal to support safe production chains for foods of animal origin. Indeed, the FEEDAP (FEED additives and substances used in Animal Production) panel of the European Food Safety Authority (EFSA) is specifically devoted to this area (3, 5).

What does it mean “safety” for a feed additive? Feed additives are at the crossroad between animals, health and the environment, The scientific criteria underlying regulatory requirements are consistent with the One Health (OH) concept: this aims to address complex issues at the human-animal-environment interface through collaboration, communication, and coordination across all relevant disciplines (6). While the OH evolved primarily to deal with zoonoses (7), it is now considered to include also the environment-animal-human interactions associated with many chemical hazards in foods of animal origin (8, 9). Consistent with the OH perspective, the EFSA assessment of substances used in feed integrates animal health and welfare, from both the standpoints of benefits (efficacy) (10) and hazards (safety) (11), as well as the assessment of toxicity (with derivation of health-based guidance values, if needed) and exposure (residues in animal products) for human consumers in order to characterize risks (12). In addition, the FEEDAP panel also assesses the safety of feed additives for users and the environment (Figure 1). Farmers and personnel of feed manufacturing facilities may be exposed through inhalation and contact with skin and mucosae (13). There are instances of feed additives (e.g., aminoacids produced by microbial fermentation) of no concern for animals and consumers, but posing a risk by inhalation to people handling the additive due to exposure to bacterial endotoxins (14); in other cases the hazard is represented by the inherent (physico)chemical properties of the additive, e.g., chemical nature or particle size. In addition, the FEEDAP Panel has to assess the exposure of the ecosystems and the potential ecotoxicological effects resulting from the passage of active substances through animal excreta into soil and water (15). Telling examples are the essential elements copper and zinc, largely added as nutritional supplements in feeds for farm animals: the FEEDAP Panel has assessed the reduction of the use levels of these trace elements in feeds to decrease the environmental impact while still preventing the risks for animal health associated with nutrient deficiency (16, 17). These two opinions developed an integrated assessment of benefits and risks for both animals and the environment, consistent with a OH approach, by comparing different options, i.e., business-as-usual vs. different levels of reduction of the maximum allowed concentrations of copper or zinc in feeds.

Alike OH, the risk–benefit assessment (RBA) of foods is a scientific effort to tackle complexity: RBA strives to develop a holistic and interdisciplinary approach to estimate the overall impact of food on health, thus to assess together the negative and positive health effects associated with consumption of foods, food components, or diets by weighing scientific evidence and uncertainties (18). Critical aspects to implement the RBA in food safety include the problem formulation with the definition of scenarios to be assessed, a tiered approach (with an initial assessment followed by potential refinements, if needed) and a common metric (e.g., Disability Adjusted Life Years - DALYs) to describe both beneficial and adverse effects (18, 19). Till recently RBA has mainly pivoted on the assessment of contaminants and nutrients in foods such as fish and seafood (20, 21); however, RBA may be useful and relevant for many different fields of food safety, and more case studies are needed to validate a sound, stepwise approach (18).

This paper presents and discusses three case studies derived from the opinions of the FEEDAP Panel in order to point out issues for extending and validating the use of RBA in other domains of food safety as well as for integrating OH considerations (e.g., animal health) into the RBA framework. One case study actually covers two subcases concerning the supplementation of feeds with essential nutrients (i.e., iodine and cobalt) that can have significant adverse effects in humans at excess intakes; the other two cases concern additives that are aimed at reducing undesirable substances in feeds that can harm human health, namely: aflatoxin binders to prevent Aflatoxin M1 contamination of milk, and formaldehyde to prevent microbial contamination of feeds.

It is to be noted that none of the EFSA opinions discussed below in relation to the three selected case studies was a formal RBA. Rather, the risk-benefit elements inherently contained in each of these assessments are outlined to show that all of the case studies qualify as pertinent items for RBA and highlight that the safety of use of feed additives can involve RBA considerations which fit into a OH perspective.

## Case Study 1: Trace Elements

### Iodine

#### RBA Question

Iodine is an essential element for thyroid function and is supplemented to all animal species. Legal limits of incorporation of iodine in feeds do exist. Nevertheless at excess exposure levels iodine can adversely affect animal and human health. In humans, excess iodine primarily affects the thyroid function and on this basis a tolerable upper intake level (UL) has been defined (22, 23).

The RBA question can then be phrased as “Should maximum allowable iodine levels in feeds be lowered to prevent excessive human exposure via food of animal origin? How this goal can be achieved without increasing the risk of deficiency in animals?”.

#### RBA

An EFSA assessment was first issued in 2005 (24); then, with different and lower regulatory limits, a second assessment was issued in 2013 (25). The scientific assumptions supporting the assessments are summarized in Table 1.

**Table 1:** Lines of evidence supporting the RBA of iodine use as nutritional additive in feeds.

Essentiality for farm animals	Iodine is an essential element for all farmed animal species (24, 25)
Factors driving the risk of iodine deficiency in farm animals	Widespread feed supplementation is warranted by low environmental iodine in several areas, presence of goitrogenic agents, greater requirements in high-producing animal categories, e.g., dairy cows (24, 25)
Impact of iodine deficiency in farm animals	Iodine deficiency, even subclinical, may significantly affect animal productivity and fertility (24, 25)
Iodine excretion in animal products	Iodine is actively excreted in milk and eggs (24, 25)
Iodine essentiality and excess in humans	Parallel to iodine deficiency, excess iodine may also affect thyroid function in humans: UL ranges from 200 μg/day for toddlers to 600 μg/day for adults (22, 23)

The first opinion (24) considered 10 mg/kg feed as the maximum regulatory limit of iodine, as iodate or iodide salts, in feeds for food-producing animals (except horses, for which the authorized level was 4 mg/kg, and fish, with authorized levels of 20 mg/kg). The assessment confirmed that in most cases iodine supplementation is necessary, due to the low basal content of feedingstuffs, especially during growth, reproduction and lactation. However the requirements were reviewed and found to be largely lower than the regulatory limits, i.e., between 0.1 and 1.1 mg/kg feed. Higher dietary iodine supply results in a limited body deposition but also in an enhanced excretion via milk and eggs. Thus, among foods from terrestrial animals milk and eggs show the highest iodine concentrations, although iodine found in milk may originate also from other sources, such as disinfectants used in milking (26). Model calculations with milk and eggs were performed with two scenarios: maximum legal limits (worst case) and levels of iodine feed supplementation in current practice (up to 4 mg/kg, realistic case). The realistic case model led to an intake of iodine corresponding to 50% of the UL in adults consuming daily the equivalents of 1.5 l of milk and 100 g of eggs; this accounted for a margin of safety considered as “sufficient” for other iodine dietary sources, such as seafood, algal-based food and supplements, and iodized salt (26–28). For younger age classes a sufficient margin of safety with the respective UL existed as well, but the food intake data suffered from significant uncertainties. Conversely, the worst case scenario showed that adults may exceed the UL with milk and egg consumption alone, without taking into account other sources. The assessment also noted significant uncertainties, including the lack of up-to-date data on iodine requirements in major food-producing species (which may have changed due to advances in breeding methods, leading to higher growth rates and milk yield) and on dose-response relationships of iodine content of foods of animal origin upon feed supplementation.

The second assessment (25) stemmed from the new regulation of iodine in feeds, where following the previous EFSA assessment, the maximum level for dairy cows and laying hens was lowered to 4 mg/kg in complete feeds. In addition, the assessment considered new evidence on dose-response of iodine deposition and, most important, improved food consumption data based on the EFSA comprehensive food consumption database. In particular, conservative figures (95th percentile, consumers only) for the intake of the main relevant food items (i.e., meat, milk and eggs) for adults and toddlers were considered. The exposure of consumers was calculated in two scenarios, one applying the authorized maximum iodine contents in feed and the other one applying reduced contents. The iodine levels in food of animal origin, if produced according to the authorized maximum content of iodine in feed, would have represented a substantial risk to high consumers of milk and, to a minor extent, of eggs. The UL for adults would have been exceeded by a factor of 2 and that for toddlers by a factor of 4. The alternative scenario entailed a reduction of the maximum iodine concentrations in feed for dairy cows and laying hens to 2 and 3 mg/kg feed, respectively, levels still meeting the requirements of animals. The exposure of adult high-consumers to iodine from food of animal origin would have been be up to 80% of the UL; however, iodine intake in high-consuming toddlers would have remained above the UL (1.6-fold).

This conclusion led to a set of related recommendations: reduce to 2 mg/kg feed the maximum iodine content in feed for dairy cows and minor dairy ruminants (sheep, goat, water buffalo); avoid the supplementation of iodine to farm animals via water for drinking because it is difficult to avoid overdosing; finally, regarding the outcome of the risk assessment in toddlers, the FEEDAP recommended monitoring the iodine status of toddlers.

Overall, in this RBA case potential risks for human health were identified due to excessive consumer exposure but the evidence-based assessment of animal nutritional requirements led to reduce the iodine supplementation levels in feeds, hence minimizing human health risks.

### Cobalt

#### RBA Question

The question can be summarized as follows: “Cobalt is a component of the essential nutrient cobalamin (vitamin B12); both inorganic Co(II) salts and cobalamin are supplemented to all animal species. Legal limits of incorporation of cobalt in feeds do exist. However, inorganic cobalt is highly toxic and, most importantly, a carcinogen by inhalation: its use can pose a risk to users. Is it possible to replace inorganic cobalt or lower supplementation levels to address the risk to users without posing a risk of deficiency to animals?”.

#### RBA

An assessment was first issued in 2009 (26), then, with different and lower regulatory limits, a new assessment was issued in 2012 (27). The assessment assumed that cobalt is an essential element only as a component of vitamin B12: deficiency of this vitamin may seriously impact growth and productivity of food-producing animals, the main signs being anemia and impaired reproduction (26). Cobalamin has a very low toxicity with no need for determining a UL for humans (22, 28); the use of feed additives based on vitamin B12 does not pose concerns for the health of farm animals or humans (29). However, inorganic cobalt provided a completely different picture; indeed, the International Agency for Research on Cancer (IARC) classified cobalt and its inorganic salts as potential carcinogens to humans (30).

The first assessment (26) considered the regulatory scenario at the time, where cobalt salts could be supplemented to all animal species, up to 2 mg/kg total cobalt (supplemental plus background) in complete feeds. The necessity of cobalt supplementation for the different farmed species was first determined. Monogastric animals (such as pigs and poultry) only require vitamin B12, not cobalt, and consequently there is no need for any cobalt supplementation. On the contrary, in ruminants cobalt is readily utilized since the ruminal microflora can synthesize vitamin B12, provided dietary cobalt is available in sufficient quantities. Actually, in these species cobalt supplementation may be more efficient and safe than vitamin B12 supplementation, due to the high ruminal degradation rate of oral vitamin B12. A comparable conclusion applies also to two minor food producing species: horses (which are consumed in some EU areas) and rabbits, where cobalt is converted to cobalamin via hindgut fermentation. Thus only ruminants, horses and rabbits need cobalt; their requirements would be amply covered by a total (background plus supplementation) cobalt level of 1 mg/kg feed. Therefore, the background cobalt concentrations naturally present in feed ingredients (usually amounting to up to 0.5 mg/kg feed) should be considered in order to establish supplementation levels within the total cobalt content of up to 1 mg/kg feed.

This first FEEDAP opinion considered that insufficient data on the oral toxicity of inorganic cobalt represented an uncertainty for the consumers risk assessment. A number of non-genotoxic adverse effects have been reported in humans, the most sensitive being polycythemia, for which a provisional daily intake of 600 micrograms/day (“minimal risk level”) was identified. However, no data were available on the potential carcinogenicity by the oral route either in humans or in experimental animals. The presence of inorganic cobalt in foods of animal origin (from both carry over from feeds and environmental or other sources) is variable among species and products, but overall very low: the intake of cobalt by foods of animal origin was estimated by the FEEDAP to be up to 14 μg/day, providing an ample margin with the provisional daily intake of 600 μg/day. The limited data in the open literature on the dietary cobalt intake in Europe were consistent with this estimate.

On the other hand, the risk for users was readily recognized as the critical safety issue. Since no specific inorganic cobalt compound was considered, exposure parameters (e.g., dusting potential) were unavailable and the FEEDAP Panel could only make a qualitative assessment. Cobalt compounds present an evident toxicity for the respiratory tree including effects for which a threshold is difficult to identify; namely, dichloride and sulfate are skin and respiratory sensitizers and carcinogenic by the inhalation route. The available evidence suggests that the carcinogenic effects of cobalt may be due to oxidative DNA damage. For non-carcinogenic pulmonary effects, the US Agency for Toxic Substances and Disease Registry has proposed a minimum risk level of 0.1 μg Co/m^3^ air (31). This first qualitative (“screening”) assessment could therefore only recognize that potential serious human health exist in regard of user exposure and indicate ways to minimize it, making avail of the evidence on animal physiology and nutrition. Thus the FEEDAP Panel recommended to restrict the use of cobalt compounds as additives to feed for ruminants, horses and rabbits; in these species cobalt supplementation should not exceed 0.3 mg Co/kg complete feed. Moreover, since people handling feeds might be exposed to inorganic cobalt naturally present as background, an exposure which is difficult to avoid, the FEEDAP Panel also recommended reducing the authorized maximum cobalt content from all sources to 1 mg/kg complete feed for all terrestrial species.

Following these recommendations, further assessments of cobalt-based feed additives considered only the use in ruminants, horses and rabbits (27). The new assessment highlighted the genotoxicity of cobalt compounds and clearly stated that exposure by inhalation must be avoided. A new consideration of issues related to consumer safety, including the remaining uncertainties on the oral carcinogenicity and the deposition in edible tissues and products, further strengthened the need for keeping the cobalt levels in feeds as low as possible without damaging animal health and productivity; thus, the new evidence confirmed the conclusions of the previous assessment.

Overall, in this RBA case potential risks for human health were identified due to a concern for inhalation exposure of users. As in the case of iodine, the evidence-based assessment of animal nutritional requirements led to reduce the supplementation levels in feeds, hence minimizing human health risks.

## Case Study 2: Aflatoxin Binders For Dairy Ruminants

### RBA Question

Aflatoxin contamination of feeds for dairy ruminants leads to the excretion of the metabolite Aflatoxin M1 in milk with consequent contamination also of dairy products such as cheese. Aflatoxin M1, although less potent than the parent compound (aflatoxin B1), has the same toxicological profile, being a liver toxicant and a potential genotoxic and carcinogenic agent (32). In 2020 the EFSA concluded that the exposure to aflatoxin M1 in milk and dairy products may pose health concerns, in particular to high consumers of the younger age groups (32). Considering also that aflatoxin contamination of feeds and the related presence of aflatoxin M1 in milk are liable to increase due to climate changes (9), several aflatoxin binders have been proposed for use as feed additives. Therefore the RBA question is “Can the use of aflatoxin binders reduce risks for human health without unexpected adverse effects?”.

### RBA

The assessment of aflatoxin binders relies on a number of scientific assumptions (32, 33) that support the potential health benefits for consumers: they are summarized in Table 2.

**Table 2:** Lines of evidence supporting the RBA of aflatoxin binders in feed for dairy ruminants.

Aflatoxin B1 as re-emerging contaminant	Aflatoxin B1 contamination of feeds for dairy ruminants is re-emerging with climate changes (9, 34)
Presence of its metabolite Aflatoxin M1 in milk and dairy products	Exposure to aflatoxin M1 may occur through milk, cheese and other dairy products, where the metabolite can concentrate as it is bound to the protein fraction of milk (32). The enrichment factors in diverse cheese types can be remarkably different and current regulatory limits may require updating (35)
Risk analysis of aflatoxin M1 in milk and dairy products	Aflatoxin M1 has similar toxicological characteristics as the parent compound. Legal limits for aflatoxin B1 in feeds and aflatoxin M1 in milk are established in the EU as a component of a farm-to-fork approach to prevent potential risks for consumers (32, 33)
Mode of action of mineral binders*^a^* to decrease aflatoxin bioavailability	Based on physicochemical properties: negatively charged and with high surface area, pore volume, swelling ability, and high cation exchange capacity. This mode of action may impact on the bioavailability of nutrients and drugs (36)
EU regulation of the use of mycotoxin binders	Only in feeds with Aflatoxin B1 levels compliant with legal limits (37–39)

a *E.g., bentonite, zeolite, montmorillonite, hydrated sodium calcium aluminosilicate*.

Clay minerals or mineral adsorbents can bind or adsorb mycotoxins in their interlayer spaces, external surface, and edges (36). Mycotoxin binders/adsorbing agents are intended to reduce to reduce aflatoxin bioavailability in complete feeds; in the European Union these additives are permitted only in complete feeds with aflatoxin levels compliant with the legal maximum tolerated limit; thus, the regulation intends to avoid that additives are used to salvage feeds that do not fulfill the safety requirements (33). Meanwhile the criteria to assess efficacy, i.e., the actual reduction of consumer's exposure are quite strict. The reduced bioavailability of Aflatoxin B1 from contaminated feeds must be demonstrated in *vivo*, through the reduction of AFM1 excretion in milk of dairy cows. A reduced bioaccessibility *in vitro* or a reduced AFM1 excretion in cows exposed to feed with AF contamination above the legal limit can only provide supportive evidence (10). Surely, since the efficacy, and hence the health benefit, of mycotoxin binders must be assessed case-by-case, *in vitro* screening methods are of use, such as adsorption assays simulating physiological pH values (40–43) or more complex models mimicking the rumen environment (44). From the - often overlooked - standpoint of safety, it is important to screen the interaction between mycotoxin binders and veterinary drugs with respect to the potential non-specific binding of drugs (45).

Mycotoxin binders may adversely affect animal health, in particular, they can interfere with the digestibility, absorption of essential elements or medications; in general these effects are more evident in pigs and poultry compared to ruminants (36, 37, 46). Actually calcium montmorillonite clay added to the diet of dairy cows challenged with aflatoxin-contaminated feed reduced the excretion of AFM1 without any effect on milk yield and the concentrations of milk fat, protein, lactose, vitamin A and riboflavin (47). A study on sheep showed no effect on fiber digestibility or nitrogen retention (48). This evidence of limited adverse effects in dairy ruminants is also supported by data on monogastric species. Beta-D-glucan biopolymers from yeast cell walls did not exert major effects on nutrient absorption in poultry (49). In the pig, montmorillonite nanocomposite did not affect serum and liver iron, copper and zinc (50). In regard of unspecific binding to veterinary antibiotics, an *in vitro* screen showed no competition of tylosin or doxycycline with AFB1 for binding to a bentonite-based mycotoxin binder (45). Interactions with doxycycline or tylosine occur, but only at concentrations in feeds >10 mg/kg (45, 51). On the other hand montmorillonite can lower the bioavailability of doxicycline (52). Clay and/or bentonite-based feed additives may contain naturally occurring dioxins, however their bioavailability and carry-over to animal products seem low (53). In the EU assessment framework of feed additives, the limiting factor for mycotoxin binders appears to be the missing demonstration of benefit at the required conditions, rather than any indication of safety concerns at the proposed conditions of use; this scenario has occurred for aflatoxin binders based on bentonite (38, 39). As far as potential hazards are concerned, *in vitro* toxicity studies on mineral adsorbents showed cytotoxic effects including oxidative stress, reduction in cell viability, apoptosis, and DNA damage (36). While the potential to induce such effects *in vivo* when the additive is mixed into feed has to investigated case by case, the available data cannot rule out a potential to alter the integrity of the digestive tract, paving the way, e.g., to the entry of pathogens.

Search for new binders is warranted by the predictable increase of the aflatoxin issue elicited by climate changes. These include natural clays and ashes from developing countries (43), yeast wall components (42, 54), agricultural by-products (40, 55), molecularly imprinted polymers (41) and nanomaterials (56). In particular, nanomaterials may operate through different modes of actions either by inhibiting the mold growth, mycotoxin adsorption, and also by reducing the toxic effect (57). However, in many cases studies on efficacy present in the literature are done on laboratory animals or *in vitro* rather than in field studies on dairy cows and no attention is given to identify potential adverse side effects. In particular for nanomaterials, the bioavailability and deposition in edible tissues and products should be determined in relation to their physicochemical characteristics; toxicological concerns derive from the potential to enter living cells and interact with their components, including DNA. This is why any nanomaterial meant to be applied in the food and feed chain, including as feed additive, has to undergo a tiered assessment exactly focusing on nanospecific risks (58).

Binders are not the only strategy to reduce health risks by Aflatoxin M1. Potential alternatives are represented by products (usually bacteria, fungi or enzymes) that target aflatoxin production by the mycotoxigenic *Aspergillus spp*., either by inhibiting aflatoxin biosynthesis or by promoting their degradation into non-toxic metabolites. These approaches may be applied to complete feeds or to feed ingredients highly liable to aflatoxin contamination, such as grains or grain-derived silages (46, 59, 60). Substances favoring detoxification, rather than binding aflatoxin and impairing its bioavailability, might avoid the potential hazards for animal health of aflatoxin binders, provided that efficacy is proven in field conditions and that aflatoxin-degradation products are actually of low toxicity. It is possible that detoxifiers alter the immunity and microbiome of the digestive tract (61). Indeed, EFSA has favorably assessed the efficacy in silage of fumonisin esterase, an enzyme degrading another mycotoxin, fumonisin; the very low toxicity of this product and its lack of persistence in feeds indicated no safety concerns (62). Thus, similar products might be envisaged for AFM1, considering the concerns raised for consumers' health due to its presence in milk and the need to find a safe and sustainable way to reduce the risks.

May a regulated use of AFM1 binders in the EU bring benefits for human health? Possibly yes: according to the recent EFSA assessment (32), the margin of exposure between the benchmark dose lower confidence limit (BMDL) for a benchmark response of 10% (BMDL_10_) of 4 μg/kg for AFM1 and the estimated intakes in EU populations were often below 10,000, especially in younger age groups, indicating that a health concern can not be ruled out. This highlights that the enforcement of legal limits for AFM1 should be integrated with other preventive measures with a farm-to-fork approach in order to minimize the human health risk (33).

A preliminary screening makes it evident that benefits of mycotoxin binders, if proven by adequate evidence, may far outweigh any potential risks. The RBA appears to critically depend on the specific binder considered, its mode of action and the potential issues that may be associated to its use. However, the use of mycotoxin binders unavoidably lead to some broader considerations that overlap with risk management.

The EU approach intends to prevent unsafe material to be recovered for use in the food chain, consistent with the general policy goal of a high level of food safety; in this framework the inherent “risk” of tools such as mycotoxin binders is to be used as replacers of good farming practices, which are a core component of the EU strategy for food safety. On the other hand, adopting a strict approach on mycotoxin binders in scenarios with a less than optimal food security, might eventually lead to wastage of resources and to a weakening of dairy chain sustainability, in particular when climate changes lead to contamination peaks (34). In such scenarios the risk managers might consider a regulated use of mycotoxin binders as an option to recover contaminated feedingstuffs.

Overall, this RBA case showed that, whereas mycotoxin risks are re-emerging with climate changes, mycotoxin binders (e.g., clays) can be effective tools to reduce the concerns due to aflatoxin M1 in milk. While mycotoxin binders should not replace good farming practices, health benefits, if proven by adequate evidence, may far outweigh potential risks.

## Case Study 3: Use of Formaldehyde as Preservative in Feedstuffs to Prevent Microbial Contamination

### RBA Question

Feed manufacturing is liable to microbiological contamination, leading to hazards for animal health and carry-over of pathogenic bacteria to foods of animal origin [e.g., *Salmonella spp*. (63)]. Formaldehyde (FA) may work as an effective and affordable biocide, but it also shows strong toxic effects, including carcinogenicity. Is the reduction of microbiological risks outweighed by risks for the target species, the consumer or the user?

### RBA

FA is an established preservative in feedingstuffs for all animal species at the proposed conditions of use, as assessed by EFSA, ranging from a minimum content of 68 mg/kg feed to a maximum content of 1,000 mg/kg feed (64–66). On the other hand, FA is a recognized carcinogen by inhalation (67, 68).

The risk associated with the use of FA as feed preservative has to be considered with regard to the exposure of the consumer, of the target species and of the user (i.e., the farm worker), according to the OH perspective. In this regard it is worth taking into account that FA is a volatile substance and that the toxicological profile of FA shows marked differences after oral or inhalation exposure. In particular, the carcinogenicity of FA after oral exposure is not demonstrated (67).

Regarding the consumer, an additional intake via feed may lead to a moderate increase of retention in edible tissues of farm animals. The available residue studies confirmed that the use of FA in feeds increased the concentrations in meat and milk, however the concentrations found in the residue studies were still in the range of the reported background concentrations in foods. Literature data indicate a contribution <20% by FA residues (from whatever source) in foods of animal origin to the overall dietary intake of FA (64, 65). Therefore, while uncertainties on the available analytical methods prevent a robust exposure assessment, data indicate that the use of FA as preservative in feedingstuffs may lead to a limited increase of residues in edible tissues and products with little impact, if any, on the overall consumer exposure. Hence, FA under the proposed conditions of use does not lead to an appreciable risk for consumers (64–66).

On the other hand, the farm worker handling the feed that contains FA as preservative, or adding FA when mixing the feed in the farm, would be exposed to FA through the respiratory route and possibly though skin and eyes. Inhaled FA has little systemic bioavailability but it is highly reactive at the contact site, forming both protein and DNA adducts. FA raises serious concerns in occupational settings as is a strong irritant, a potent skin and respiratory sensitiser (also associated with occupational asthma) and a proven human carcinogen by the respiratory route (67, 68). In the EU, short-term occupational exposure limits for FA based on irritation have been recommended. However, no safe level for long-term exposure of skin, eyes and respiratory system could be established as FA is a potent sensitiser and no threshold for tumor induction could be identified, as the mechanism of carcinogenesis is unclear (64, 65). Due to the strong reactivity of FA with proteins and DNA, the carcinogenic action of FA at the contact site (nasopharyngeal and sinonasal cancers) can be due to a direct genotoxic effect, to non-genotoxic events associated to chronic irritation or to a combination of both mechanisms (64, 65). The carcinogenicity of FA is recognized by international agencies. FA is classified as a carcinogen 1B (presumed to have carcinogenic potential for humans) by the European Chemicals Agency (ECHA) (67), based on limited evidence of carcinogenicity in humans (mainly nasopharyngeal tumors) and sufficient evidence from animal studies. The IARC considered FA as carcinogenic to humans (group 1), based on sufficient evidence in humans, with a causal association with both nasopharyngeal tumors and leukemia (65).

Local irritation is expected to strongly promote FA carcinogenesis. The demonstration of a uniquely non-genotoxic mode of action would theoretically allow the setting of a threshold for carcinogenesis; however non-irritant local concentrations of FA are known to produce DNA adducts, a lesion causally related with genotoxic carcinogenicity. Considering that the contribution of FA adducts to carcinogenicity cannot be ruled out, a threshold for cancer induction cannot at present be envisaged (64, 65).

Target species are exposed through the oral route, but, considering the volatility of FA, also respiratory exposure has to be considered relevant. The safety of FA for target species was investigated in tolerance studies in poultry, piglets and calves. While zootechnical parameters, hematology and clinical biochemistry were not affected by the additives in poultry, Japanese quail and piglets, adverse effects of FA on reproductive organs were seen at 930 mg/kg feed for male poultry and at 1,850 mg/kg feed for female Japanese quail. Since the maximum use concentration in feeds is 680 mg/kg, the margin of safety for poultry used in reproduction (including laying hens) is in the range 1.5–2.5, therefore relatively low. In addition, whereas FA induces reproductive toxicity in avian species (65), the safety in reproducing farm animals could not be established due to the lack of studies. No tolerance studies in cattle were available, but severe gross- and microscopic lesions of the alimentary tract compatible with clinical symptoms were recorded in calves fed formalin-treated skimmed milk; therefore no safe concentration can be established for veal calves (64, 65).

FA fulfills the classical requirements for a preservative additive, as it exhibits the potential to inhibit microbial growth and to prevent recontamination for a certain time. However, while FA has the potential to reduce microbial growth in an already contaminated feed at a concentration of 200 mg/kg complete feed, its efficacy in the prevention of recontamination requires considerably higher concentrations. Moreover, the evidence toward the benefits of the use of FA on the hygienic quality of feeds shows limitations in regard of zoonotic pathogens. In particular, experimental studies showed an effect on *E. coli*, although only at 380 mg/kg feed and above, i.e., well above the minimum recommended use concentration. Conversely, the effect on *Salmonella typhimurium* was not consistently shown and the efficacy against *Campylobacter jejuni* could not be demonstrated (65). Overall, the benefits in preventing the contamination by major zoonotic bacteria were less evident than expected. Moreover, it should be noted that the reduction of microbial load in contaminated feed is not sufficient to guarantee the feed safety, as residual bacterial toxins and endotoxins may still be present in the feed (66).

In conclusion, although the use of FA as a preservative would not pose an additional risk for the consumer, a safe level for all animal species and categories could not be identified. Furthermore, the use of FA as feed additive would imply a risk for the farm worker, associated with respiratory, skin and eye exposure and would at least require appropriate protective measures in order to minimize exposure, yet considering that a threshold cannot be established. Meanwhile the evidence of a beneficial impact on feed hygiene shows significant limitations in regard of the control of zoonotic agents. Overall, the potential risks associated with the use of FA as feed preservative would far outweigh the possible health benefits.

## Discussion and Conclusions

The case studies presented in this paper illustrate that the safety assessment of feed additives can involve RBA considerations. These have to fit into the existing regulatory framework, which – at least in the EU – involves an assessment of benefits and risks for animal health, the safety for consumers of products from treated animals, occupational health (safety for users) and the safety for the environment. Hence, this risk assessment framework builds in an OH-based perspective, although OH is not explicitly mentioned as a regulatory basis.

As in other RBA applications to different food-related domains, one key challenge highlighted by the cases presented is how to approach the R/B comparison. In the case of feed additives, due to the above-mentioned OH framework, R and B may concern different species (farm animals and humans, in some cases also the environmental biota). Meanwhile, ensuring animal health and nutrition translates into impacts relevant to human health and welfare, i.e., improved livestock productivity and availability of food of animal origin for humans, hence improved food security. This also means provision of specific nutrients, including vitamins and trace elements (e.g., iodine in milk and dairy products). Finally, improved animal health entrains a reduced risk of zoonoses and a reduced need for using antibiotics, with a reduced selective pressure toward antimicrobial resistance (1, 69). Thus, safe and effective feeds, including feed additives, may have substantial benefits for human health, although such benefits are complex to model and quantify.

In turn, occupational and environmental health aspects, such as those encountered with several feed additives, see the case studies of cobalt and FA, entail sustainability considerations. Noticeably, other assessments exist where the impacts of different scenarios on animal health and nutrition and on the ecosystems have been compared, as in the cases of copper (16) and zinc (17). Thus, the case of feed additives may often differ from the classical RBA cases (e.g., nutrients and contaminants in seafood) (20, 21, 70), where risks and benefits can be compared using a common “currency.”

All the above aspects have been highlighted as avenues for the further development of RBA, which evolved from considering the human health impact of food intake scenarios as the endpoint but is nowadays called to consider and balance the direct health impact with health-relevant effects on other factors such as environmental sustainability, food security, and societal values (18).

According to the core framework, each RBA compares two or more scenarios. In the cases of nutritional additives (16, 17, 24–27) the scenarios are the different levels of presence in feeds. In the cases of risk-reducing additives (37–39, 64–66), the scenarios refer to a risk-risk comparison encompassing the “business-as-usual” scenario and that resulting from the use of the feed additive.

In the cases considered in the present paper, the EFSA assessments of feed additives would correspond to RBA stopping at the first tiers, i.e., at step 1 (initial assessment) or step 2 (refinement of the assessment) according to the guidance on human health risk-benefit assessment of food of the EFSA Scientific Committee (19). Indeed, a qualitative screening for each scenario is sufficient in most cases since the outcome is either the identification of a clear advantage or the existence of safety concern in terms of animal, human and/or ecosystem health (see the case of formaldehyde with reference to animal and human health). A refinement of the assessment, comparing different options was needed in the cases of cobalt and iodine. In such cases, a residual risk may be identified also in the most favorable scenario, such as the slight exceedance of UL in high-consumers toddlers in the iodine case study (25): this was communicated to risk managers in a transparent way.

The ‘screening nature' of the assessments performed for feed additives avoided the major issue of finding a common currency for modeling risks and benefits, which is required for the full development of RBA involving solely human health (18) and corresponds to step 3 in the guidance of the EFSA Scientific Committee (19). The OH framework of the feed additives entrains comparisons between apples and oranges such as between animal nutrition (and food security) and human health (24–27) or environment (16, 17). On the other hand, in the case of feed additives the problem formulation - the RBA question - can be usually dealt within the first, screening step of the RBA process, i.e., whether a health advantage (e.g., in terms of risk reduction) can be demonstrated at use level(s) without a significant health concern. In other cases, the risk(-benefit) assessors present the outcomes of different scenarios to risk(-benefit) managers in order to support decision making.

Feed additives are regulated substances that in most cases are supported by dossiers produced by applicants to fulfill regulatory requirements. Nevertheless, relevant uncertainties may be encountered when looking at FEEDAP opinions: one example observed in our case studies is the carcinogenicity mechanism of FA, which could determine whether it is possible to define a “tolerable level of inhalation exposure” (63–65). Other examples of uncertainties potentially relevant to a RBA are a comprehensive appraisal of the impact of mycotoxin binders on animal nutrition (37) and the assessment of different levels of copper in feeds in regard of the contribution to the development of antibiotic resistance in animal gut and the environment (16).

Feed composition is involved in a core topic and starting point of RBA, namely the combined presence of nutrients (e.g., omega 3 fatty acids, iodine) and contaminants (methylmercury, bioaccumulating endocrine disruptors such as dioxins, PCBs and PBDEs) in fish and seafood; in several scenarios risks may outweigh benefits (20). Farmed fish, representing a growing fraction of the global fish consumption, does not present significant differences with wild fish in regard of the contents of nutrients and contaminants when considering differences due to species and location (70). The replacement of the conventional ingredients in aquaculture feeds, based on fish-derived proteins and oils, with vegetable-based feed ingredients can drastically reduce the accumulation of the main contaminants in farmed fish (71). The seafood content of nutrients may also be modulated by feeds: recent research shows that the use of algal ingredients can enrich the iodine content of rainbow trout (*Oncorhynchus mykiss*), a freshwater fish low in iodine, while minimizing the bioaccumulation of mercury (72). The nutritional profile of fish modified by the new scenarios might deserve more attention in the RBA; for instance, the use of vegetable oils can significantly modulate the lipid profile in fish flesh, depending on the oil and fish species (71), e.g., resulting in a decrease of omega 3 fatty acids (73, 74). The novel, vegetable-based, aquaculture feeds were reported to cause a lowering of the fat-soluble vitamin D3 in fish, supporting a request to EFSA to assess the safety of higher addition levels of this vitamin, with the goal of improving both animal and human nutrition (75). The assessment represented an interesting example of RBA with “neutral” outcome: it recognized that the increasing use of plant-based feed materials in aquaculture feeds could induce a decrease in vitamin D3 content in feedingstuffs; however, there was no evidence that the current total (background + supplemented) maximum EU content of vitamin D3 could cause any appreciable risk of deficiency in salmonids, as the best investigated fish group. Also in regard of human nutrition and safety, notwithstanding many uncertainties, there was no evidence that increasing the level of vitamin D3 in feedingstuffs may lead to an important increase of the vitamin in fish flesh; therefore, the increased supplementation will lead neither to exceeding the UL, even in high consumers, nor to a significant contribution to reduce the human deficiency (75).

Transparency is important in risk assessment and even more in RBA, which entails a higher level of complexity. Under this respect, it is worthwhile to mention the paper by Fjaeran and Aven (76): pivoting on a FEEDAP opinion as a case study, the authors highlight the importance of the “trust” and “distrust” components in the role of risk assessment. Assessors can act to improve conditions of trust by adopting an understanding of risk, stressing uncertainty and knowledge aspects when conceptualizing and characterizing risk; according to the authors, acknowledging the existence of societal “distrust” toward risk assessment and responding to it, can become a resource in order to build a “critical trust.”

RBA can become highly relevant in specific instances of the assessment of regulated substances, such as feed additives, as shown in the present paper. The RBA conceptual framework may need adaptations to the terms of reference for regulated substances. Meanwhile, the framework may help a consistent approach in the relevant cases, starting from the formulation and interpretation of the question.

## Author Contributions

AM: supervision, conceptualization, writing—original draft preparation, and writing—review and editing. GA and FM: writing—original draft preparation and writing—review and editing. FC: writing—original draft preparation, writing—review and editing, and visualization. All authors contributed to the article and approved the submitted version.

## Conflict of Interest

The authors declare that the research was conducted in the absence of any commercial or financial relationships that could be construed as a potential conflict of interest.

## Publisher's Note

All claims expressed in this article are solely those of the authors and do not necessarily represent those of their affiliated organizations, or those of the publisher, the editors and the reviewers. Any product that may be evaluated in this article, or claim that may be made by its manufacturer, is not guaranteed or endorsed by the publisher.
